# Cold denaturation as a tool to measure protein stability

**DOI:** 10.1016/j.bpc.2015.05.007

**Published:** 2016-01

**Authors:** Domenico Sanfelice, Piero Andrea Temussi

**Affiliations:** a MRC National Institute for Medical Research, The Ridgeway, London, UK; b Dipartimento di Chimica, Universita' di Napoli Federico II, Napoli, Italy

**Keywords:** Thermal resistance, Thermal stability, Cold denaturation, Stability curve

## Abstract

Protein stability is an important issue for the interpretation of a wide variety of biological problems but its assessment is at times difficult. The most common parameter employed to describe protein stability is the temperature of melting, at which the populations of folded and unfolded species are identical. This parameter may yield ambiguous results. It would always be preferable to measure the whole stability curve. The calculation of this curve is greatly facilitated whenever it is possible to observe cold denaturation. Using Yfh1, one of the few proteins whose cold denaturation occurs at neutral pH and low ionic strength, we could measure the variation of its full stability curve under several environmental conditions. Here we show the advantages of gauging stability as a function of external variables using stability curves.

## Highlights

• Protein stability is not described adequately by unfolding temperature.
• The area under the stability curve offers a better assessment of stability.
• Cold denaturation indirectly offers a simple way to calculate the stability curve.
• Ambiguous cases of protein stability are resolved by area measurement.

## Graphical abstract

**Figure:**
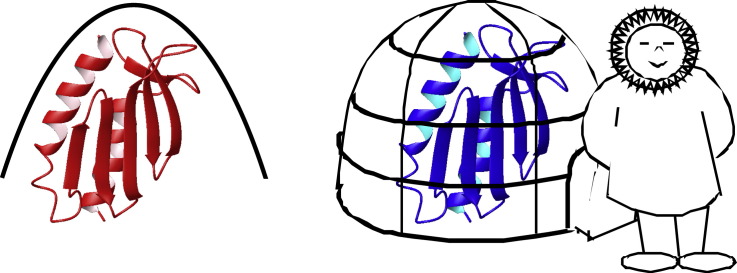

## 1 Introduction

Native proteins are often said to be just marginally stable because the thermodynamic stability of folded species of many proteins is only 1 to 4 kcal/mol higher than that of the corresponding unfolded species. This status appears of critical importance whenever stability is affected by environmental factors which can influence the function of proteins.

Thermal stability of proteins may seem an almost elusive concept, because it is difficult to account for thermodynamic stability and thermal resistance at the same time. The first term is generally defined as the difference in free energy between the folded and unfolded species (Δ*G*), whereas the second one is described by the melting temperature (*T_m_*). Both terms can be judged from an examination of the protein stability curve [1], but it is not always easy to calculate stability curves for a protein under all experimental situations.

Reliable methods to study protein stability are therefore of paramount importance. The experimental approach generally used to gauge protein thermal stability is to monitor the change in some property of the protein as it unfolds. The most common techniques employed are spectroscopies of various nature, e.g. circular dichroism, fluorescence or NMR. In the case of thermal unfolding, the variation of the chosen property as a function of temperature generates thermograms whose fit can yield both the midpoint of unfolding (*T_m_*) and the enthalpy change at the same temperature (Δ*H^o^_Tm_*) with good accuracy. The latter parameter is related to the change in free energy (Δ*G*), the measure of thermodynamic stability. *T_m_* and Δ*G* measure thermal resistance and thermodynamic stability respectively [2]. When used to describe protein stability, they both have shortcomings but the main problem is that in a general case there is very little correlation between them. It is common place to use the variation of melting temperature (Δ*T*) as a faithful measure of stability, implying proportionality with thermodynamic stability (Δ*G*). Indeed, Becktel and Schellman [1] in their exhaustive analysis of the protein stability curve showed that when the change in free energy is *small* and the melting temperatures are not too close to the temperature of maximum stability (*T_S_*) it is possible to relate the change in melting temperature to the value of the perturbation free energy at the melting temperature. However, they also warned that the relationship between changes in melting temperature and changes in free energy is linear only if we can make the assumption that the portions of the two stability curves chosen to measure the temperature increment (Δ*T*) behave as parallel straight lines crossing the abscissa (*T*). In actual cases this is often not true: a common cause of failure stems from the simultaneous change of the enthalpy of folding and of heat content (Δ*C_p_*). Any change in the curvature of the curve (− Δ*C_p_/T*) will make the two portions not parallel.

Another serious limitation in the use of high temperature melting to assess protein stability is that heating a protein solution is not always a reversible process. The most common cause of irreversibility resides in the onset of aggregation phenomena, mainly driven by hydrophobic interactions that are most relevant at high temperatures. We have shown that access to the whole stability curve can help to circumvent this problem [2].

Unfortunately, the fit of the thermograms is totally insensitive to the value of the heat capacity difference between the native and denatured states (Δ*C_p_*), a parameter we must know to calculate the stability curve [1] as can be seen from equation (1):

(1) $$\Delta G=\Delta H_{m}\left(1-\frac{T}{T_{m}}\right)+\Delta C_{p}\left\{\left(T-T_{m}\right)-T\;\ln\left(\frac{T}{T_{m}}\right)\right\}\text{.}$$

Szyperski et al. [3] have shown that if for a given protein it is possible to observe both cold and heat denaturation, the fitting of the thermogram becomes quite sensitive to the value of Δ*C_p_* and thus the procedure leads to a reliable determination of the stability curve. We show here that comparisons of stability based on the whole stability curve can shed light on trends otherwise difficult to follow.

## 2 Cold denaturation

Protein unfolding caused by heating a protein solution from room temperature to higher values is a familiar phenomenon and is simply referred to as “thermal denaturation” whereas unfolding caused by cooling the protein from room temperature to lower values is called “cold denaturation”.

Thanks mainly to the work of Privalov and coworkers cold denaturation is now a firmly established concept [4]. However, one has often the impression, when referring to it, that cold denaturation is an outcast of protein chemistry, either because some researchers simply do not know it or because it is considered an exotic subject. Is there something fundamentally unique behind cold denaturation? No, this property is a simple consequence of the stability curve which, being an arched curve, crosses the zero point of free energy at two points, one at temperatures lower than room temperature and another above room temperature, defining two unfolding transition points. The main problem is that for most proteins cold denaturation occurs at temperatures well below water freezing. This is precisely the reason why cold denaturation is rarely observed in wild type proteins. A minor problem, in our opinion, may be psychological: heat denaturation is accepted intuitively because it is common experience that heating can degrade many systems. On the other hand, unfolding by “cold” is counter-intuitive because lowering the temperature generally just slows down processes and eventually stabilizes the systems.

The most common approach to overcome the difficulty poised by the limited accessibility of very low temperatures is to raise the midpoint of cold denaturation above the temperature of water freezing, by destabilizing the protein either via *ad hoc* mutations and/or by adding denaturants to the solution. The drawback of all approaches which lower protein stability is that, by definition, they prevent any study on the influence on the wild type protein of other external factors. During the past few years we have observed that the cold denaturation of yeast frataxin (Yfh1) occurs at temperatures higher than water freezing and in solutions whose composition is consistent with physiological conditions [5]. This is why the cold denaturation of Yfh1 has been dubbed “unbiased”. Yfh1 belongs to the frataxin family, whose human ortholog is involved in the neurodegenerative disease known as Friedreich's ataxia [6].

**Fig. 1:**
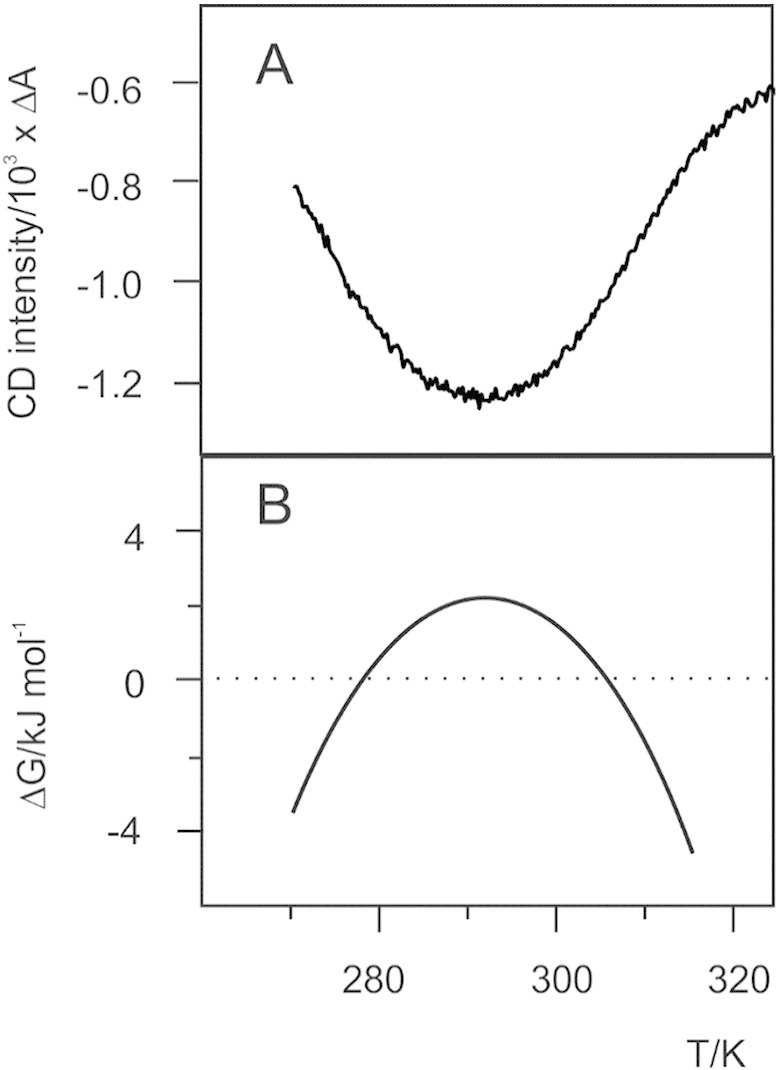
Comparison between the thermal denaturation curve of Yfh1 and the corresponding stability curve. (A) Plot of CD signals at 222 nm of Yfh1 in HEPES at pH 7.0 as a function of temperature. (B) Stability curve of Yfh1 in HEPES at pH 7.0. A constant *ΔCp* of 1.8 kcal mol^− 1^ K^− 1^ was used in this case [5].

Even a glance at Fig. 1 may suffice to explain why a set of spectroscopic data covering both cold and heat denaturation (Fig. 1A) is very sensitive to the curvature of the stability curve (− ΔCp/T) and thus ideally suited to calculate the whole protein stability curve (Fig. 1B). Analysis of the stability curve yields all relevant thermodynamic parameters connected with unfolding. This study [5] suggested that Yfh1 can be used as a model system to study protein stability in several environmental conditions. In particular, we have used it to study the influence of alcohols, the relative importance of crowding and confinement and the difference between ionic strength and specific salt effects.

## 3 Alcohols: typical denaturants?

Alcohols are generally regarded as typical protein denaturants. However, there were sparse hints that, these molecules might not have adverse effects on protein stability, at least at low temperatures and moderate concentrations [7], [8]. We investigated the effect of methanol, ethanol and trifluoro-ethanol, three alcohols of increasingly hydrophobic side chain on the stability of Yfh1, our ideal model system [9].

**Fig. 2:**
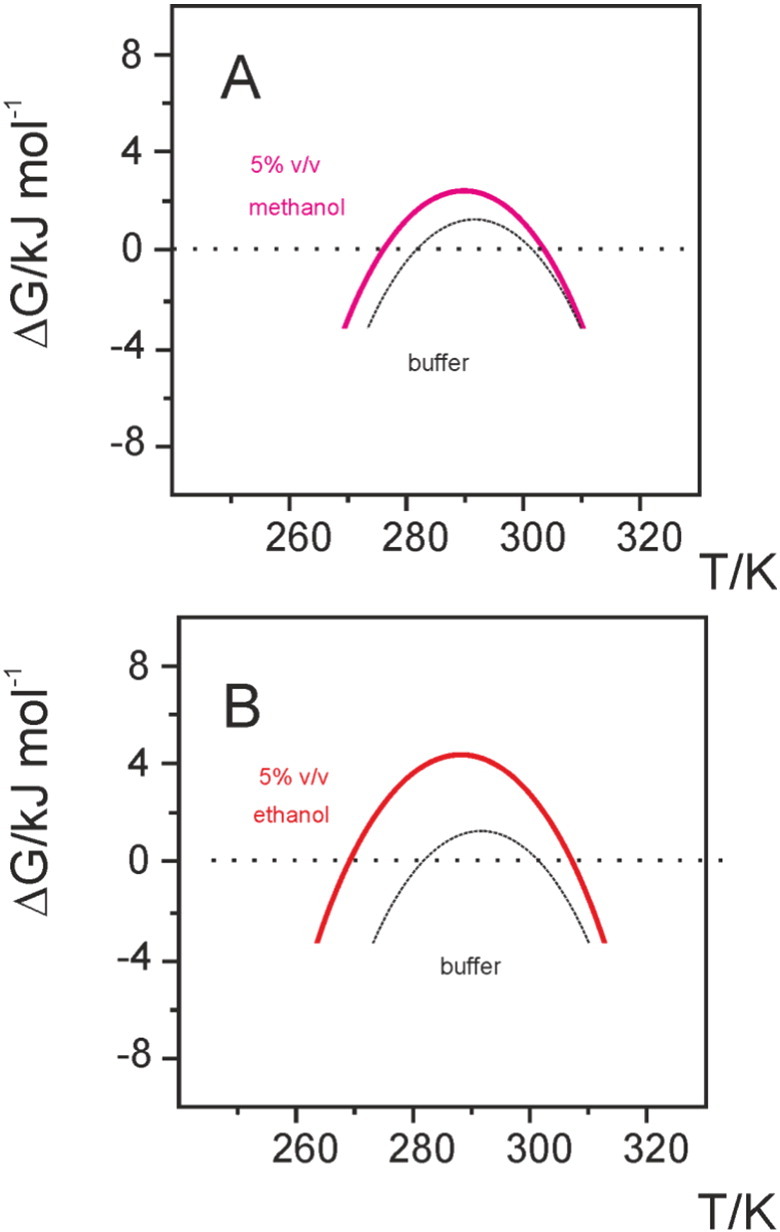
Stabilization of Yfh1 induced by modest amounts of methanol or ethanol. A) Comparison of the stability curves of Yfh1 in buffer (dashed curve) and in a buffered solution containing 5% v/v of methanol (solid curve). B) Comparison of the stability curves of Yfh1 in buffer (dashed curve) and in a buffered solution containing 5% v/v of ethanol (solid curve). The values of *ΔCp* are 1.9 kcal K^− 1^ mol^− 1^ for buffer, 1.7 kcal K^− 1^ mol^− 1^ for 5% methanol and 5% ethanol [5].

All alcohols employed were able to influence the CD thermograms but, surprisingly, the effect of methanol on the heat denaturation temperature is minimal whereas the cold denaturation temperature is greatly affected (Fig. 2A). Such behavior might seem paradoxical at first site, because, if we use thermal resistance as the only criterion for stability, the high temperature melting point (*T_m_*) would hint at a marginal or decrease of stability induced by alcohols whereas the marked decrease of the low unfolding temperature (*T_c_*) suggests a considerable stabilization. The overall effect on protein stability can only be estimated from a comparison of the stability curves, particularly by assessing the area covered by different curves. The comparisons reported in Fig. 2 show that even modest alcohol concentrations, of the order of ca. 5% v/v, lead to a very large increase in the area under the stability curve. This increase is consistent with the stabilization hinted at by the decrease of the low unfolding temperature (*T_c_*), although it seems to contradict the insensitivity of thermal resistance.

An important consequence of the study on the influence of alcohols on the stability of model system Yfh1 is that, on the basis of its results, it becomes possible to interpret old literature data which appeared ambiguous and difficult to understand.

Velicelebi and Sturtevant [10] had observed that addition of alcohols to lysozyme solutions did produce a small decrease in the unfolding temperature (*T_m_*) as expected in the case of a denaturant but, at the same time also an increase of the enthalpy of unfolding (*ΔH_m_*) and a decrease of *ΔC_p_*. In the light of the results on Yfh1 it is possible to interpret their results as an enlargement of the stability curve and a simultaneous shift of the whole curve towards low temperatures. Similar results were obtained by Fu and Freire [11] for cytochrome C. In both cases, the decrease of thermal resistance heralded by a decrease of *T_m_* is paralleled by a lowering of *T_c_* and by an increase of the free energy at the maximum of the stability curve [9]. As a result the area under the stability curve between *T_m_* and *T_c_* increases greatly. Altogether these results point to a stabilization of lysozyme and cytochrome C by alcohols, at least at moderate alcohol concentrations.

## 4 Crowding and confinement

Owing to the presence of many macromolecules, the interior of cells has been described as crowded and/or confining. The two words, although often considered synonyms, are not interchangeable: crowding is dynamic in nature while confinement describes a static situation [12]. Both crowding and confinement are expected to increase the stability of folded proteins with respect to the corresponding unfolded species, on the assumption that volume exclusion ought to favor more compact conformations. Although recent evaluations seemed to suggest that the effects of crowding on protein stability are modest [13], [14], it must be emphasized that it has been always difficult to compare the effects of crowding and confinement, mainly because most studies have been performed on different proteins.

The availability of a friendly system like Yfh1 prompted us to make a detailed comparison [15]. As a crowder we employed a well-known synthetic polymer, Ficoll 70, which is widely regarded as neutral, whereas the chosen confining agent was polyacrylamide gel (PAG), previously used for the same protein [16].

**Fig. 3:**
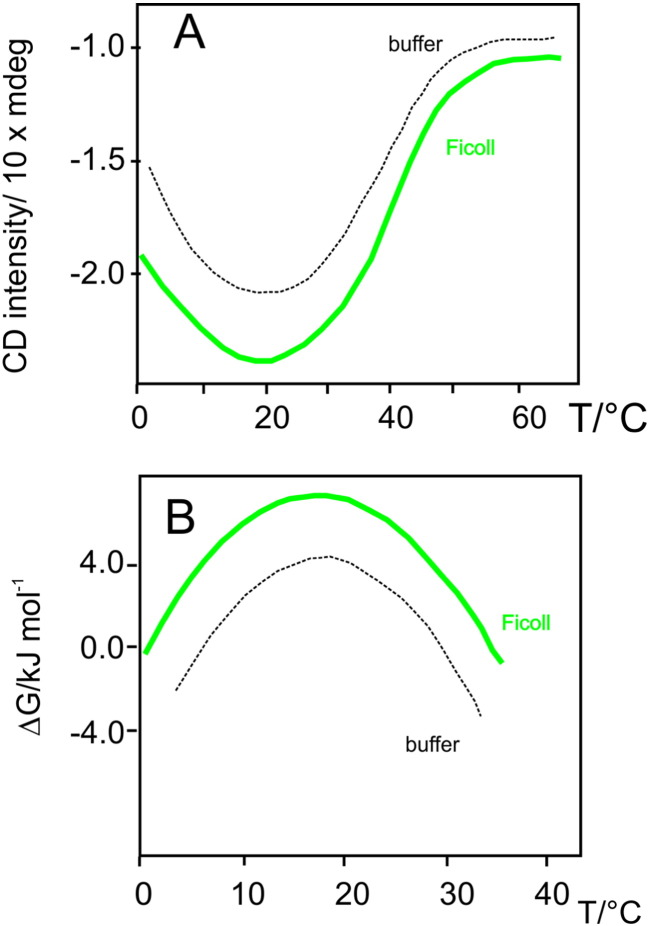
Comparison between the CD thermograms of Yfh1 and the corresponding stability curves with and without Ficoll 70. A) CD intensity of Yfh1 at 222 nm as a function of temperature. The dashed curve corresponds to a solution in 20 mM HEPES buffer at pH 7.4. The solid curve corresponds to a solution in 20 mM HEPES buffer at pH 7.4 containing 15% w/w of Ficoll 70. B) Stability curves for HEPES (dashed curve) and 15% w/w Ficoll 70 (solid curve) derived from the thermograms of panel A. The values of *ΔCp* are 1.8 kcal mol^− 1^ K^− 1^ for buffer and 1.4 kcal mol^− 1^ K^− 1^ for 15% w/w Ficoll 70 [15].

The behavior in the presence of Ficoll could be studied by CD spectroscopy because Ficoll does not have a CD signal interfering with that of the protein. As shown in Fig. 3, the crowding experiment confirmed that the synthetic crowder Ficoll 70, even at the moderate concentration of 15% w/v is rather efficacious in stabilizing the folded form of Yfh1: we observed an increase of *Tm* but also an increase in enthalpy (Δ*H*) and a decrease in heat capacity difference between the native and denatured states (Δ*C_p_*).

The comparison of crowding and confinement for the same system was made difficult by technical limitations: PAG is not transparent at the CD wavelength used to monitor unfolding and the quantitative use of 2D NMR is intrinsically problematic. At low temperature in the confined environment of PAG the T_2_ relaxation time is abnormally influenced when the viscosity of the solution is high and yields unreliable values of peak areas in the 2D spectrum. However, in the less viscous buffer solution it was possible to measure stability curves consistent with those derived from CD data of the same solution. Thus we knew that we could rely on the more conventional high temperature melting points in the three environments. The values obtained are consistent with those obtained by CD in the presence of Ficoll and also in good agreement with those previously measured in the same environment by fluorescence spectroscopy [16]. We concluded that both crowding and confinement have a fairly large influence on the stability of Yfh1 [15].

## 5 Specific salt effects

The stability of Yfh1 is extremely sensitive to the presence of salts [17]. This circumstance is linked to the role played by this protein in the biosynthetic machinery which produces iron–sulfur clusters [18]. However, when studied in the framework of the biosynthesis of clusters, it was not clear whether the effect of different ions could be ascribed to either two intrinsically different mechanisms, particularly for monovalent cations, which act in a nonspecific way by increasing ionic strength, as opposed to divalent cations, which bind specifically to sites important for the function of frataxins. We undertook an exhaustive stability study using a variety of monovalent cations and a few selected divalent cations. CD thermograms obtained in the presence of salts yielded stability curves that clarify the issue.

**Fig. 4:**
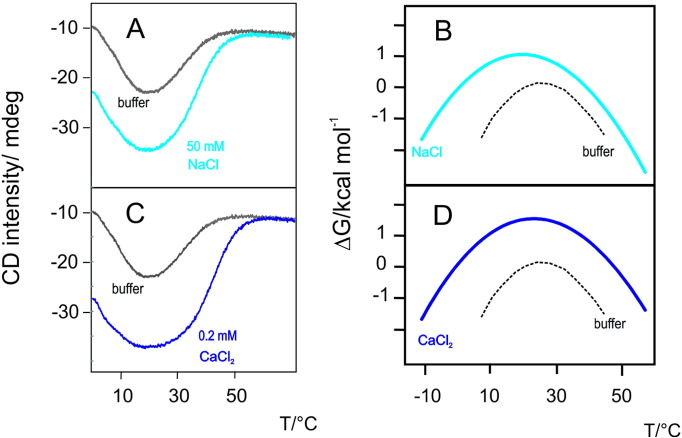
Comparison of the effects of NaCl and CaCl_2_ on the stability of Yfh1. A) CD intensity of Yfh1 at 222 nm in 20 mM HEPES buffer at pH 7.4 as a function of temperature. The dashed curve corresponds to a buffered solution. The solid curve corresponds to a buffered solution containing 50 mM NaCl. B) Stability curves for HEPES (dashed curve) and 50 mM NaCl (solid curve) derived from the thermograms of panel A. C) CD intensity of Yfh1 at 222 nm in 20 mM HEPES buffer at pH 7.4 as a function of temperature. The dashed curve corresponds to a buffered solution. The solid curve corresponds to a buffered solution containing 0.2 mM CaCl_2_. D) Stability curves for HEPES (dashed curve) and 0.2 mM CaCl_2_ (solid curve) derived from the thermograms of panel C. The values of *ΔCp* were 3.0 kcal K^− 1^ mol^− 1^ for buffer, 1.6 kcal K^− 1^ mol^− 1^ for NaCl and 1.6 kcal K^− 1^ mol^− 1^ for CaCl_2_[19].

Although all salts stabilize the folded form of Yfh1, the stabilizing effect of divalent cations manifests itself at concentrations orders of magnitude lower than those needed when employing monovalent cations [19]. As an example, a comparison of the effects of NaCl and CaCl_2_ is reported in Fig. 4. Panels A and C show the comparisons of the thermograms obtained from monitoring CD intensity at 222 nm in a range of temperature from 0 °C to 60 °C under the influence of sodium chloride or of calcium chloride, for panels A and C respectively. In both cases the salt leads to a stabilization of the folded species, as indicated by the large increase of the absolute value of CD intensity, but the calculation of the corresponding stability curves makes the stability increase more evident.

### 5.1 Panels B and D show the stability curves corresponding to the thermograms of panels A and C

It is clear that the two salts induce a similar stabilization of the folded form of Yfh1. However, for calcium chloride it is only sufficient to reach a concentration of 0.2 mM whereas the concentration of sodium chloride that induces a comparable stabilization is 50 mM, i.e. 250 times larger.

## 6 Conclusion

As pointed out in the introduction, and emphasized by Pucci et al. [20], stability is indeed a dual concept, comprising both “thermodynamic stability” and “thermal resistance”. The main problem in using the two concepts is that there is usually very little correlation between “thermodynamic stability” and “thermal resistance” to the point that they can yield contradictory answers. A paradigmatic example of ambiguous cases is furnished by the influence of moderate alcohol concentrations described above.

In this review we have shown that the best way to judge the stability of a protein under different environmental situations is to compare stability curves. Exploiting the unique features of yeast frataxin (Yfh1) we have shown that it is possible to unveil surprising properties of alcohols, once considered typical denaturants, whereas they stabilize several proteins at low temperature and moderate concentrations. Yfh1 proved a system of great value also to compare the relative efficiency of crowding and confining conditions and the relative influence of cations of different charges to stabilize the native fold.

It is tempting to generalize our approach by proposing the use of stability curve as the most reliable way to compare protein stability. The main drawback of this approach is the difficulty to measure the heat capacity difference between the native and denatured states (Δ*C_p_*). This task is greatly facilitated when it is possible to observe cold denaturation above water freezing, but we would like to emphasize the use of the stability curve as a general method even if it is not always easy to observe unbiased cold denaturation. Even when it is only possible to observe the beginning of the second transition, the asymmetry of the reversible thermal unfolding transition may be sufficient to extract Δ*C_p_* accurately from the curve fitting process. In addition, the fitting of spectroscopic thermograms is not the only method to evaluate Δ*C_p_*. In all cases for which it is feasible to obtain a reliable measure of Δ*C_p_* it will be possible to calculate the stability curve and thus evaluate stability reliably.
